# Life years lost associated with mental illness: A cohort study of beneficiaries of a South African medical insurance scheme

**DOI:** 10.1016/j.jad.2023.08.013

**Published:** 2023-08-06

**Authors:** Yann Ruffieux, Anja Wettstein, Gary Maartens, Naomi Folb, Cristina Mesa-Vieira, Christiane Didden, Mpho Tlali, Chanwyn Williams, Morna Cornell, Michael Schomaker, Leigh F. Johnson, John A. Joska, Matthias Egger, Andreas D. Haas

**Affiliations:** a Institute of Social and Preventive Medicine, University of Bern, Bern, Switzerland; b Division of Clinical Pharmacology, Department of Medicine, University of Cape Town, Cape Town, South Africa; c Medscheme, Cape Town, South Africa; d Graduate School of Health Sciences, University of Bern, Bern, Switzerland; e Department of Sociology, Ludwig-Maximilians-Universität München, Germany; f Centre for Infectious Disease Epidemiology & Research, School of Public Health, University of Cape Town, Cape Town, South Africa; g Department of Statistics, Ludwig-Maximilians-Universität München, Germany; h HIV Mental Health Research Unit, Neuroscience Institute, University of Cape Town, Cape Town, South Africa; i Division of Neuropsychiatry, Department of Psychiatry and Mental Health, Faculty of Health Sciences, University of Cape Town, Cape Town, South Africa; j Population Health Sciences, Bristol Medical School, University of Bristol, Bristol, UK

**Keywords:** Excess mortality, Life years lost, Mental disorders, South Africa

## Abstract

**Background:**

People with mental illness have a reduced life expectancy, but the extent of the mortality gap and the contribution of natural and unnatural causes to excess mortality among people with mental illness in South Africa are unknown.

**Methods:**

We analysed reimbursement claims from South African medical insurance scheme beneficiaries aged 15–85 years. We estimated excess life years lost (LYL) associated with organic, substance use, psychotic, mood, anxiety, eating, personality, developmental or any mental disorders.

**Results:**

We followed 1,070,183 beneficiaries for a median of three years, of whom 282,926 (26.4 %) received mental health diagnoses. Men with a mental health diagnosis lost 3.83 life years (95 % CI 3.58–4.10) compared to men without. Women with a mental health diagnosis lost 2.19 life years (1.97–2.41) compared to women without. Excess mortality varied by sex and diagnosis, from 11.50 LYL (95 % CI 9.79–13.07) among men with alcohol use disorder to 0.87 LYL (0.40–1.43) among women with generalised anxiety disorder. Most LYL were attributable to natural causes (men: 3.42, women: 1.94). A considerable number of LYL were attributable to unnatural causes among men with bipolar (1.52) or substance use (2.45) disorder.

**Limitations:**

Mental diagnoses are based on reimbursement claims.

**Conclusions:**

Premature mortality among South African individuals with mental disorders is high. Our findings support interventions for the prevention, early detection, and treatment of physical comorbidities in this population. Targeted programs for suicide prevention and substance use treatment, particularly among men, can help reduce excess mortality from unnatural causes.

## 1. Introduction

Mental illness is a leading and increasing cause of disease burden globally (GBD 2019 collaborators, 2022). In South Africa, the total number of disability-adjusted life years attributable to mental illness nearly doubled from 550,000 to 929,000 over the past 30 years (GBD 2019 collaborators, 2022). Mental disorders now rank among the top ten leading causes of disease burden in South Africa (GBD 2019 collaborators, 2022). A survey conducted in the country, using diagnostic interviews, reported a lifetime prevalence of mental disorders of 30 % (Herman et al., 2011). This figure likely underestimates the true lifetime prevalence due to recall bias (Moffitt et al., 2010).

Although most mental disorders do not lead directly to death, they increase the risk of suicide, accidental death, and premature mortality from physical illness (Chesney et al., 2014; De Hert et al., 2013; Liu et al., 2017; Richmond-Rakerd et al., 2021; Walker et al., 2015). Higher rates of premature mortality from physical illness can be attributed to a higher incidence of physical comorbidities among people with mental illness and worse access to or engagement in health care (De Hert et al., 2013, 2011). Physical illnesses can also increase the risk of mental illness due to various biological and psycho-social mechanisms (Prince et al., 2007).

Meta-analyses of studies—mainly from high-income countries—demonstrated that people with mental illnesses, particularly those with severe conditions, have more than double the mortality rates of the general population and die on average 10 years prematurely (Chesney et al., 2014; Walker et al., 2015). Due to differences in life expectancy and disease burden, estimates from high-income countries cannot be generalised to low- and middle-income settings. However, evidence from low- and middle-income settings is scarce (Chesney et al., 2014; Walker et al., 2015). In Ethiopia, mortality in people with schizophrenia was six times higher than in the general population and three to four times higher in those with major depression (Teferra et al., 2011; Mogga et al., 2006). Another study from Ethiopia found that people with severe mental disorders, including schizophrenia, bipolar disorder, and severe depression, died 28 years earlier than the general population (Fekadu et al., 2015). Among people living with HIV in South Africa, those diagnosed with a mental illness had a three times higher mortality risk compared to those without diagnoses (Haas et al., 2020).

We aimed to investigate excess mortality among people with mental illness in South Africa. We analysed longitudinal reimbursement claims and vital registration data of beneficiaries of a large South African medical insurance scheme. We calculated excess life years lost (LYL) and adjusted hazard ratios (HRs) for excess mortality among people diagnosed with a mental illness. This study is one of Africa’s most comprehensive analyses of excess mortality among people with mental illness.

## 2. Methods

### 2.1. Study design and participants

We analysed longitudinal reimbursement claims and vital registration data of a cohort of beneficiaries of a large South African medical insurance scheme. Reimbursement claims included outpatient claims, hospital claims, and pharmacy claims. Outpatient and hospital claims contained International Classification of Diseases, tenth revision (ICD-10) diagnoses made in outpatient and hospital settings. Pharmacy claims contained information on active drug ingredients coded according to the Anatomical Therapeutic Chemical (ATC) classification system, drug strength, dispensed amount, and dispensed date. The vital registration system records deaths occurring at all ages. All data sources were accessible for the entire study period, spanning from January 1, 2011, to June 30, 2020. For our analysis, we included beneficiaries aged 15–84 years who were covered by medical insurance at any point between January 1, 2011, and June 30, 2020. We excluded individuals with unknown age and sex, as well as those whose vital status could not be ascertained based on the linkage to the National Population Register (NPR) (appendix, fig. S1). The Human Research Ethics Committee of the University of Cape Town and the Cantonal Ethics Committee of the Canton of Bern granted permission to analyse these data.

### 2.2. Procedures

Our primary exposures were ICD-10 diagnoses of mental disorders made in outpatient or hospital settings. We grouped diagnoses into organic mental disorders (ICD-10 codes F00-F09), substance use disorders (F10–F17, F19), psychotic disorders (F20–F29), mood disorders (F30–F39), anxiety disorders (F40–F48), eating disorders (F50), personality disorders (F60–F69), developmental disorders (F80–F89), or any mental disorder (F00–F99). We further subdivided substance use disorders into alcohol use disorder (F10) and drug use disorders (F11–F17, F19), mood disorders into bipolar disorders (F31) and depressive disorders (F32–F33, F34.1), and anxiety disorders into generalised anxiety disorder (F41.1) and post-traumatic stress disorder (F43.1). We did not analyse behavioural syndromes associated with physiological and physical factors (F50-F59) and behavioural and emotional disorders with onset usually occurring in childhood and adolescence (F90-F98) given the heterogeneous nature of these categories and, for the latter category, given the limited follow-up time and low mortality rates in young beneficiaries.

In secondary analyses, we only analysed ICD-10 diagnoses made in a hospital setting and psychiatric medication prescriptions as proxies for mental disorders. We grouped psychiatric medication following the ATC classification system into substance use medication (N07B), antipsychotic (N05A), antidepressant (N06A), anxiolytic (N05B), or any psychiatric medication (N05A, NO5B, N06A, and N07B).

We ascertained the vital status of beneficiaries based on mortality records from NPR and the medical insurance database. If the death dates recorded by the medical insurance did not match NPR data, we used death dates from NPR. Finally, we divided mortality by natural, unnatural, or unknown causes of death. Unnatural causes include all deaths by external causes, such as suicides, homicides, accidents, medical errors, alcohol intoxications and drug overdoses (ICD10 V01—Y98) and natural causes include all causes of death from chapters 1 to 18 of the ICD-10 (Statistics South Africa. Republic of South Africa, 2017). Unknown causes include death under investigation at the time of link- age, death due to unidentified causes, and death recorded only by the medical insurance but not by the NPR.

### 2.3. Statistical analysis

For each individual, we defined baseline as either the date of enrolment in the medical insurance scheme, their 15th birthday, or January 1, 2011, whichever occurred later. We followed people from baseline to the end of insurance coverage, death, their 85th birthday, or June 30, 2020, whichever occurred first. We assessed the distribution of sex, baseline age, and baseline calendar year for participants with and without mental health diagnoses.

We estimated LYL of people with mental health diagnoses and psychiatric medication. LYL measures the average difference in life expectancy among people with mental health diagnoses between the dates of diagnoses and their 85th birthday, compared to people of the same age and sex without mental health diagnoses (Andersen, 2017; Erlangsen et al., 2017; Plana-Ripoll et al., 2020, 2019). Individuals were considered unexposed until receiving a first mental health diagnosis and exposed from the date of their first diagnosis of the mental illness of interest. Using a competing risks model, we disaggregated LYL by cause of death (natural, unnatural, or unknown) (Andersen, 2013). We produced 95 % CI for the LYL estimates using bootstrap simulation. Most mental health diagnoses were recorded multiple times because ICD-10 codes are submitted with each outpatient and hospital claim. In sensitivity analyses, we restricted analysis of LYL to persons with a single ICD-10 diagnosis of a mental disorder that could result from a coding error. In another sensitivity analysis, we did not censor follow-up at the end of insurance coverage if the person died within 1 year of the end of insurance coverage to account for deaths occurring after losing insurance coverage.

Using multivariable Cox proportional hazard models, we estimated HRs with 95 % CIs for associations between each exposure and mortality from all causes, natural causes, and unnatural causes. When modeling natural and unnatural causes, we estimated cause-specific hazard ratios, censoring individuals who had died from the competing cause on their date of death (Schuster et al., 2020). All Cox models were adjusted for the exposure of interest (time-updated variable with the individual switching from unexposed to exposed at the time of the first diagnosis of the mental illness of interest), baseline age (categories 15–24, 25–39, 40–54, 55–74, 75–84), and calendar period (time-updated by categories January 1, 2011–December 31, 2013; January 1, 2014–December 31, 2016; January 1, 2017–March 14, 2020; March 15–June 30, 2020). We chose the March 15, 2020, cut-off for the calendar period to coincide with the beginning of the COVID-19 pandemic in South Africa. We fitted separate models adjusting and not adjusting for psychiatric comorbidity. Models adjusted for psychiatric comorbidity included binary indicators for organic mental disorders, substance use disorders, psychotic disorders, mood disorders, anxiety disorders, behavioural syndromes associated with physiological disturbances and physical factors, personality disorders, developmental disorders, and behavioural and emotional disorders with onset usually occurring in childhood and adolescence. In addition, we tested for interactions between sex and each exposure. We estimated HRs separately for each sex and with both sexes combined while adjusting for sex. We tested the proportional hazards assumption using a test based on Schoenfeld residuals and visual inspection of log-log plot of the survivor functions. Because the proportional hazards assumption was violated for several exposures, we performed additional analyses where we split analysis time at one- and two-year marks after the start of their time-at-risk and produced an adjusted HR for each interval.

We performed statistical analyses using R 4.1.2 (R Foundation for Statistical Computing, Vienna, Austria) and Stata (Version 16. College Station, TX: StataCorp). We used the R package *lillies* to compute LYL (Plana-Ripoll et al., 2020).

## 3. Results

We followed 1,070,183 people (517,305 men and 552,878 women) for a median duration of 3.0 years [interquartile range (IQR) 1.2–6.1) (appendix, Fig. S1). The characteristics of people with and without mental health diagnoses are shown in Table 1. During follow-up, 282,926 beneficiaries (26.4 %) received a mental health diagnosis (Table 2). Among individuals with a mental health diagnosis, there were 10,964 deaths (3.9 %), while among individuals without a mental health diagnosis, there were 21,195 deaths (2.7 %). The proportion of beneficiaries who received mental health diagnoses was higher among women (30.5 %) than men (22.1 %). The most common mental health diagnoses were anxiety disorders (16.6 %), including generalised anxiety disorder (3.0 %) post-traumatic stress disorder (1.3 %) and mood disorders (14.6 %), including depressive disorders (14.1 %) and bipolar disorder (1.8 %). The proportion of beneficiaries diagnosed with organic, substance use, psychotic, eating, developmental, and personality disorders was below 1 % (Table 2). Psychiatric comorbidity was common. For example, among beneficiaries diagnosed with a psychotic disorder, 75.2 % had also been diagnosed with a mood disorder (appendix, Fig. S2). A total of 45,579 beneficiaries (4.3 %) received a mental health diagnosis in a hospital (appendix, Table S1). Over one-third of the study population was prescribed a psychiatric medication (34.8 %), including antidepressant (22.3 %), anxiolytic (22.1 %), antipsychotic (9.5 %), and substance use (0.5 %) medication (appendix, Table S2).

**Fig. 1.**
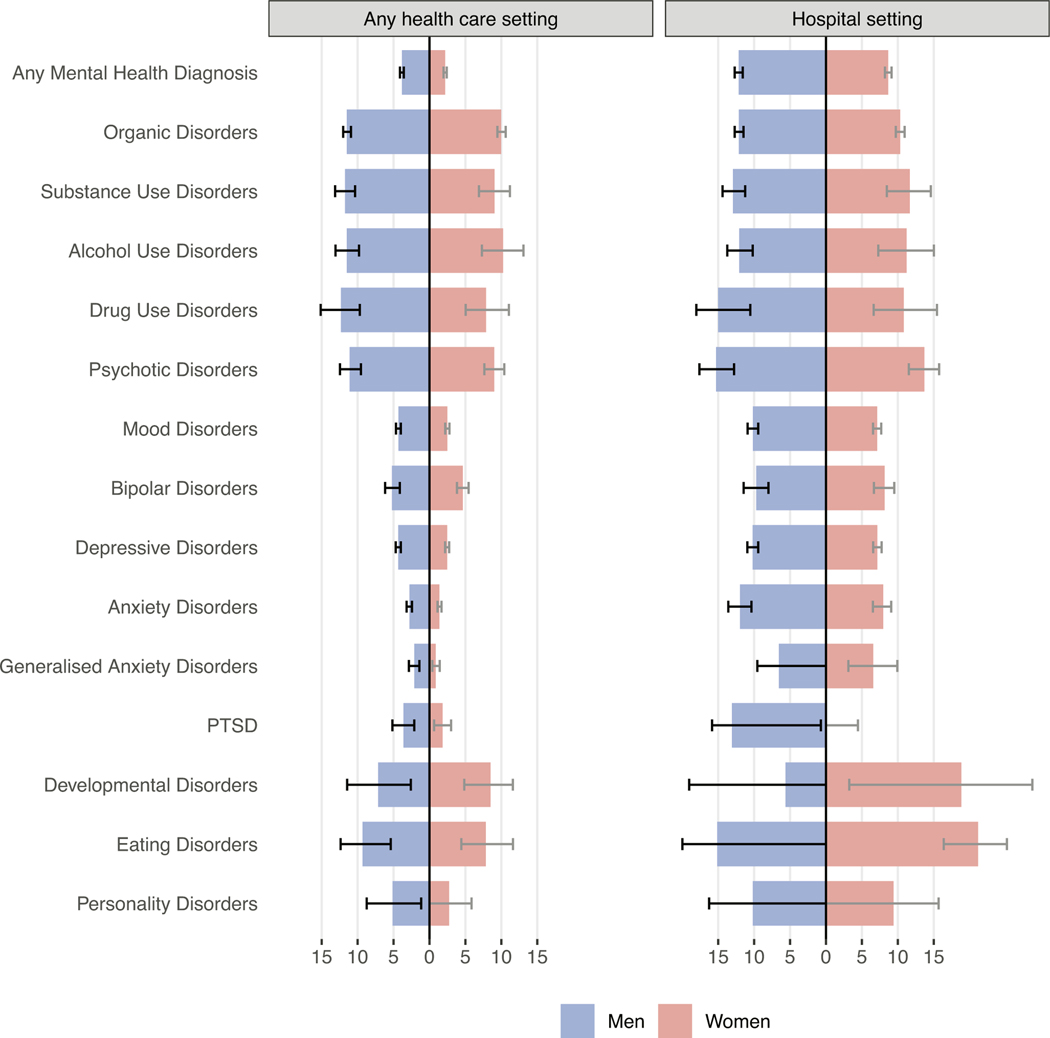
Excess life years lost among people who received mental health diagnoses compared with people of the same sex without mental health diagnoses. Data are stratified by sex and health care setting. Left panel shows data of people diagnosed in any health care setting, such as outpatient or hospital. Right panel shows data from people diagnosed in hospitals. Error bars represent 95 % confidence intervals. Confidence limits with negative values are truncated at 0 in the figure. PTSD: Post-traumatic stress disorders.

Fig. 1 shows LYL among men and women who received mental health diagnoses in any health care setting (left panel) or hospital (right panel) compared to beneficiaries of the same sex and age without mental health diagnoses. Men had higher LYL than women for all types of mental illnesses except developmental disorders. For beneficiaries diagnosed in any health care setting (outpatient or hospital), life expectancy after diagnosis of any mental disorder was 3.83 years (95 % CI 3.58–4.10) shorter for men and 2.19 years (1.97–2.41) shorter for women when compared to beneficiaries of the same age and sex without mental health diagnoses. The life expectancy of men diagnosed with an organic mental disorder, alcohol use disorder, drug use disorder, or psychotic disorder was reduced by over 10 years. Women with organic or alcohol use disorder had a similar mortality gap of 10 years. Psychotic and drug use disorders among women and developmental and eating disorders for both sexes were associated with an LYL of 7 to 10 years. Women with common mental disorders showed lower excess mortality, for example: 0.87 (95 % CI 0.40–1.43) LYL for generalised anxiety disorder and 2.47 (2.17–2.74) LYL for depressive disorders.

When considering diagnoses from hospital settings only, LYL associated with mental health diagnoses increased to 12.15 years (95 % CI 11.59–12.70) among men and 8.67 (95 % CI 8.20–9.13) among women (Fig. 1, right panel, appendix Table S3). Women hospitalised with an eating disorder (21.17 LYL, 95 % CI 16.38–25.18) and men hospitalised with a psychotic disorder (15.29 LYL, 95 % CI 12.79–17.61) showed the largest mortality gap.

Most LYL were attributable to natural causes (Tables 3): in men, 3.42 (95 % CI 3.17–3.70) LYL associated with any mental health diagnosis were from natural causes, 0.45 (95 % CI 0.32–0.59) from unnatural causes, and −0.04 (95 % CI −0.11–0.03) from unknown causes; in women, 1.94 (95 % CI 1.73–2.15) LYL were from natural causes, 0.22 (95 % CI 0.15–0.28) were from unnatural causes, and 0.03 (95 % CI −0.02–0.09) were from unknown causes. The LYL from natural causes remained higher than the LYL from unnatural causes across all types of mental illnesses and across both sexes. In comparison to other mental illnesses, a larger excess mortality burden was attributable to unnatural causes with alcohol use disorders (LYL 2.49, 95 % CI 1.41–3.60), drug use disorders (LYL 2.05, 95 % CI 0.58–3.77), and bipolar disorders (LYL 1.52, 95 % CI 0.90–2.17) among men. Among women with drug use disorder 1.85 (95 % CI 0.34–3.69) LYL were attributable to unnatural causes.

Use of psychiatric medication was associated with 4.05 LYL (95 % CI 3.79–4.12) among men and 2.26 LYL (95 % CI 2.05–2.48) among women (appendix, Table S4). When limiting exposures to beneficiaries with only one mental health diagnosis, LYL increased to 5.61 years (95 % CI 5.17–6.10) among men and 4.41 (95 % CI 3.96–4.84) among women (appendix, Table S5). Including deaths occurring within 1 year after an individual’s insurance plan ended did not markedly change results (appendix, Table S6).

**Fig. 2.**
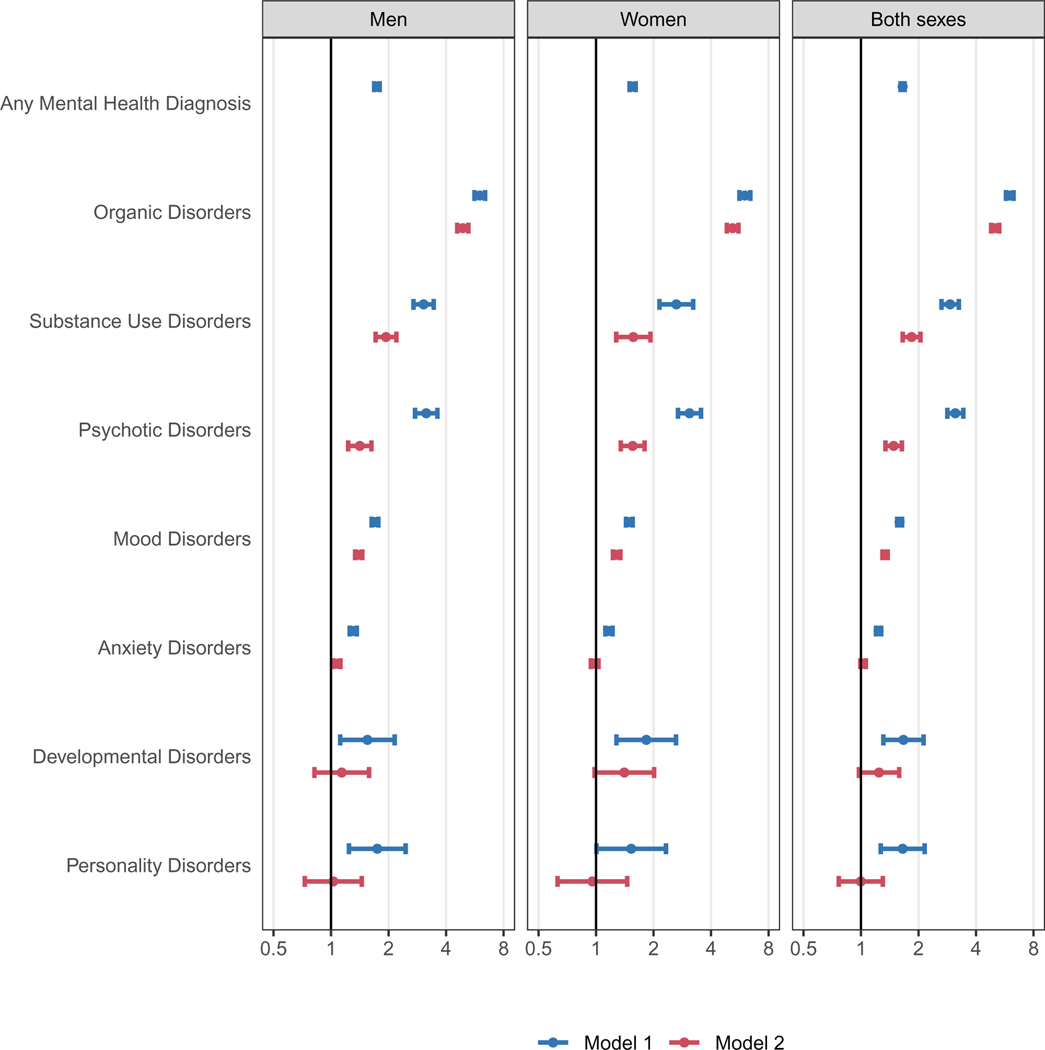
Adjusted hazard ratios comparing all-cause mortality among people with and without mental health diagnoses. We adjusted all models for baseline age, and time-updated calendar year. We adjusted models combining both sexes for sex. Model 1 does not adjust for psychiatric comorbidity; Model 2 adjusts for psychiatric comorbidity. The x-axes are on a logarithmic scale.

Fig. 2 shows adjusted HR comparing all-cause mortality of people with and without organic, substance use, psychotic, mood, anxiety, developmental, personality or any mental disorder. The mortality rate was 65 % higher among people diagnosed with mental disorders than those without mental health diagnoses (HR 1.65, 95 % CI 1.61–1.69). In models adjusted for age, sex, and calendar year (model 1), the greatest increase in mortality risk was among people diagnosed with organic mental disorders (HR 6.01 [95 % CI 5.74–6.29]), followed by those diagnosed with psychotic (3.12 [2.83–3.43]), substance use (2.93 [2.64–3.25]), developmental (1.67 [1.31–2.12]), personality (1.65 [1.27–2.15]), mood (1.59 [1.55–1.65]), and anxiety (1.24 [1.20–1.27]) disorders. After further adjusting for psychiatric comorbidity (model 2), associations were attenuated. For example, the HR for psychotic disorders decreased to 1.48 (95 % CI 1.34–1.64) and anxiety disorders were no longer associated with all-cause mortality (HR 1.03, 95 % CI 0.99–1.06). Excess mortality associated with any mental health diagnosis decreased with follow-up time: from an adjusted HR of 1.96 (95 % CI 1.82–2.10) in year 1 of follow-up to an adjusted HR of 1.60 (95 % CI 1.56–1.65) beyond the second year of follow-up (appendix, Table S7). Mental health diagnoses of most types were associated with a higher risk of natural and unnatural deaths (appendix, Table S8). Mental health diagnoses during hospitalisations were associated with a 279 % increase in mortality risk (HR 3.79, 95 % CI 3.66–3.93) (appendix, Table S9). Psychiatric medications were associated with a 77 % increase in mortality risk (HR 1.77, 95 % CI 1.73–1.81) (appendix, Table S10).

## 4. Discussion

In this cohort of South African medical insurance beneficiaries, 22 % of men and 31 % of women received mental health diagnoses over a median follow-up period of three years. On average, the remaining life expectancy after a mental health diagnosis was 3.8 years shorter for men and 2.2 years shorter for women compared to people without mental health diagnoses. The difference in life expectancy varied by sex and disorder. Eating disorders, developmental disorders, psychotic disorders, substance use disorders, and organic mental disorders were strongly associated with LYL, especially among men. In contrast, women with anxiety or depression had lower excess mortality. Most excess deaths among people with mental illness were attributable to natural causes.

To our knowledge, this study presents the most comprehensive analysis of excess mortality among people with mental illness in Africa. We compared mortality between 280,000 people with and 785,000 people without mental health diagnoses. Earlier studies of excess mortality mainly included people with severe mental illness managed in tertiary care hospitals (Walker et al., 2015). Our study included people with a broad spectrum of disorders managed at all levels of care. We ascertained mortality by linking data from a medical insurance scheme to the South African civil registration system. According to a validation study, the mortality linkage should have identified about 95 % of adult deaths (Johnson et al., 2015). The mortality data included cause of death information, allowing us to disaggregate excess mortality estimates into deaths attributable to natural and unnatural causes. Finally, we used international diagnostic criteria for the full spectrum of mental disorders to evaluate excess mortality among people with various mental health conditions while adjusting for psychiatric comorbidity.

Although the Global Burden of Disease (GBD) study recognises mental disorders as leading causes of disease burden, it likely underestimates their true burden (GBD 2019 collaborators, 2022; Vigo et al., 2016). Most mental disorders do not directly lead to death but increase the risk of suicide, accidents, and physical illness, indirectly contributing to premature mortality (Chesney et al., 2014; De Hert et al., 2013; Liu et al., 2017; Walker et al., 2015) In the GBD framework, mortality indirectly attributable to mental disorders is attributed to the cause directly leading to death as recorded on death certificates (Charlson et al., 2015; GBD 2019 collaborators, 2022) For example, suicide resulting from depression will be coded as injury and no mortality burden will be attributed to depression (Charlson et al., 2015; GBD 2019 collaborators, 2022). As a result, the GBD’s disease burden estimates may not reflect the mortality attributable to mental disorders and hence underestimate the true burden of mental disorders (GBD 2019 collaborators, 2022; Vigo et al., 2016).

We calculated an alternative metric, LYL, to quantify excess mortality among people with mental illness. LYL is a descriptive measure of excess mortality reflecting the direct and indirect causal contributions of mental disorders to premature mortality, but also the effect of non-causal confounding factors, for example, precarious living conditions or physical comorbidities influencing both the risk of a mental disorder and mortality. The metric quantifies the average difference in life expectancy after diagnoses of health conditions compared with people of the same age and sex without such diagnoses. LYL are useful for identifying vulnerable populations at high risk of premature mortality who might benefit from targeted interventions to reduce excess mortality. However, LYL must not be interpreted as reflecting the causal effect of mental disorders on mortality. Causal inference analyses are needed to quantify the mortality burden attributable to mental disorders.

Although most mental disorders were associated with substantial excess mortality, our estimates were generally lower than those reported in previous studies. A meta-analysis of studies primarily from high-income countries estimated that people diagnosed with a mental disorder died about ten years prematurely (Walker et al., 2015). Differences in overall life expectancy between these high-income settings and South Africa may partly explain our lower estimates of excess mortality associated with mental illness. Additionally, the relatively low upper age limit of 85 years used in our study for the LYL computation, compared to the 95 years used in studies of high-income countries, might also contribute to the differences observed in our results. We lowered the upper age limit to 85 years as a result of the lower life expectancy in South Africa and the fact there were few beneficiaries above that age threshold in our study. The overrepresentation of people with severe mental illness in earlier work might partly explain the difference between estimates from those studies and our work. Many studies included in the meta-analysis relied on medical records from tertiary care settings, including a large proportion of people with psychotic disorders and other severe mental health conditions associated with a high risk of premature mortality. In contrast, our study included a more representative sample of people with mental illness. We identified most mental health diagnoses based on medical records from outpatient settings. Common mental health conditions such as depression and anxiety disorders accounted for most mental health diagnoses. We included people with psychotic disorders and those hospitalised with mental disorders in our study, but persons with severe mental illness were not overrepresented. A register-based cohort study from Denmark found a lower (10.3 %) prevalence of mental illness that from our study (26.4 %), despite a longer median follow-up (Plana-Ripoll et al., 2019), which may suggest that study is not capturing milder episodes of mental illness.

People diagnosed with mental illness were at higher risk of death from natural and unnatural causes. Yet, almost 90 % of LYL associated with mental disorders were attributable to death from natural causes. Previous studies support this finding. For instance, the aforementioned study from Denmark attributed 70 % (men) and 80 % (women) of excess mortality burden to natural causes, including circulatory, respiratory, and alcohol-related liver disease (Plana-Ripoll et al., 2019). A community-based cohort study from rural Ethiopia attributed 75 % of deaths among people with severe mental illness to natural causes, mainly due to infectious diseases (Fekadu et al., 2015). Higher rates of physical illnesses among people with mental illness and poorer health care contribute to premature death from natural causes among people with mental illness (De Hert et al., 2013, 2011; Liu et al., 2017). Experts recommend interventions for reducing the burden of physical illness among people with mental health conditions to close the mortality gap (Liu et al., 2017). Interventions can target lifestyle and behaviours to prevent physical illness or early detection and appropriate treatment of common physical comorbidities through screening or collaborative or integrated mental and physical health care models (Liu et al., 2017). Furthermore, interventions aiming to reduce stigma towards mental illness among health care providers should be considered (Carrara et al., 2021).

For some disorders, unnatural causes of death accounted for considerable excess mortality. For example, among men with alcohol use disorder, drug use disorder, or bipolar disorder, unnatural causes of death accounted for about 17–29 % of total LYL. Suicide prevention, substance use treatment, and harm reduction programmes are needed to reduce excess mortality from unnatural causes (Liu et al., 2017; Tlali et al., 2022).

Men with mental illness had higher excess mortality than women with mental illness. For alcohol and bipolar disorders, sex disparities in excess mortality are at least partly attributable to higher mortality from unnatural causes among men than women. For other disorders, lower health care utilisation rates among men compared with women (Seedat et al., 2009) arising from the gendered nature of health services creating health care barriers for men (Cornell et al., 2021; Dovel et al., 2020), harmful masculine norms (Vogel et al., 2011), self-stigmatizing beliefs (Latalova et al., 2014) or differences in coping strategies (Oliver et al., 2005) may contribute to higher excess mortality for men than women. Further work is needed to design, implement, and evaluate strategies addressing men’s physical and mental health care needs.

Our study has several limitations. First, we assessed beneficiaries’ mental health status based on ICD-10 diagnoses from reimbursement claims; thus, we missed beneficiaries with undiagnosed mental disorders who may be less severely ill than those diagnosed, and beneficiaries with mental disorders who did not present for treatment. Furthermore, mental health diagnoses might be affected by diagnostic or administrative errors, although diagnoses from administrative data generally have a high positive predictive value for research diagnoses (Davis et al., 2016). Sensitivity analysis of people with only one, possibly erroneous, mental health diagnosis showed high excess mortality, suggesting this population might constitute people with untreated conditions rather than false positive cases. Second, we did not consider remissions and may have misclassified the person-time of individuals in remission. This could have led to an underestimation of the excess mortality, particularly for depression or anxiety. Third, although mortality from external causes is reliably recorded in South Africa’s vital registration system (Matzopoulos et al., 2015), data on the exact causes of death are limited. This prevented us from disaggregating mortality from unnatural causes into suicides and other causes, and mortality from natural causes into different disease categories. Fourth, our study only included data from a private-sector medical insurance scheme. Thus, our findings do not necessarily apply to people accessing the public health care sector. People using public health services generally have lower socioeconomic status. They may be at higher risk of experiencing poor mental health outcomes than employed and insured people who access private services (Hassim et al., 2007). In addition, access to mental health care in South Africa’s public sector is limited and mental disorders often remain untreated (Ruffieux et al., 2021).

In conclusion, our study demonstrates a considerable burden of premature death among people with mental illness primarily attributable to death from natural causes. People diagnosed with eating, developmental, psychotic, substance use, and organic mental disorders—especially men and those hospitalised with these conditions—are at the highest risk of premature death. These findings support implementing interventions for prevention, early detection, and appropriate treatment of physical comorbidities among people with mental illness. In addition, suicide prevention and substance use treatment programmes are needed to reduce excess mortality from unnatural causes among men with bipolar and substance use disorders.

## Supplementary Material

1

## Figures and Tables

**Table 1:** Characteristics of people who did and did not receive mental health diagnoses before their end of follow-up.

	Received mental health diagnoses	Without mental health diagnoses	Total
	*N* = 282,926	*N* = 787,257	*N* = 1,070,183

Age at baseline,			
years			
15–19	32,015 (11.3 %)	118,048 (15.0 %)	150,063 (14.0 %)
20–29	49,797 (17.6 %)	173,373 (22.0 %)	223,170 (20.9 %)
30–39	69,255 (24.5 %)	181,991 (23.1 %)	251,246 (23.5 %)
40–49	59,665 (21.1 %)	136,445 (17.3 %)	196,110 (18.3 %)
50–59	41,652 (14.7 %)	101,600 (12.9 %)	143,252 (13.4 %)
60–69	18,831 (6.7 %)	49,003 (6.2 %)	67,834 (6.3 %)
70–84	11,711 (4.1 %)	26,797 (3.4 %)	38,508 (3.6 %)
Median [IQR]	38.5 [28.5, 50.3]	35.2 [25.4, 48.5]	36.1 [26.3, 49.0]
Sex			
Male	114,377 (40.4 %)	402,928 (51.2 %)	517,305 (48.3 %)
Female	168,549 (59.6 %)	384,329 (48.8 %)	552,878 (51.7 %)
Calendar year at			
baseline			
2011–2013	188,817 (66.7 %)	411,711 (52.3 %)	600,528 (56.1 %)
2014–2016	44,244 (15.6 %)	134,390 (17.1 %)	178,634 (16.7 %)
2017–2019	47,144 (16.7 %)	211,178 (26.8 %)	258,322 (24.1 %)
2020	2721 (1.0 %)	29,978 (3.8 %)	32,699 (3.1 %)
Died^a^			
Natural cause	9662 (88.1 %)	17,978 (84.8 %)	27,640 (85.9 %)
Unnatural cause	938 (8.6 %)	2240 (10.6 %)	3178 (9.9 %)
Unknown cause	364 (3.3 %)	977 (4.6 %)	1341 (4.2 %)

Data are n (%) unless otherwise stated. IQR interquartile range.

a Denominators for percentages are total deaths in that group.

**Table 2:** Numbers and proportions of people who received mental health diagnoses during follow-up by sex.

	Men	Women	Total
	*N* = 517,305	*N* = 552,878	*N* = 1,070,183

Any mental health diagnosis (F00-F99)	114,377 (22.1 %)	168,549 (30.5 %)	282,926 (26.4 %)
Organic mental disorders (F00-F09)	3863 (0.7 %)	4655 (0.8 %)	8518 (0.8 %)
Substance use disorders (F10-F17, F19)	5275 (1.0 %)	2042 (0.4 %)	7317 (0.7 %)
Alcohol use disorders (F10)	2757 (0.5 %)	938 (0.2 %)	3695 (0.3 %)
Drug use disorders (F11-F17, F19)	2947 (0.6 %)	1226 (0.2 %)	4173 (0.4 %)
Psychotic disorders (F20-F29)	2228 (0.4 %)	2324 (0.4 %)	4552 (0.4 %)
Mood disorders (F30-F39)	57,038 (11.0 %)	98,835 (17.9 %)	155,873 (14.6 %)
Bipolar disorders (F31)	6786 (1.3 %)	12,078 (2.2 %)	18,864 (1.8 %)
Depression (F32-F33, F34.1)	54,755 (10.6 %)	95,785 (17.3 %)	150,540 (14.1 %)
Anxiety disorders (F40-F48)	67,599 (13.1 %)	110,405 (20.0 %)	178,004 (16.6 %)
Generalised anxiety disorders (F41.1)	11,521 (2.2 %)	21,062 (3.8 %)	32,583 (3.0 %)
Post-traumatic stress disorders (F43.1)	5362 (1.0 %)	8593 (1.6 %)	13,955 (1.3 %)
Developmental disorders (F80-F89)	2353 (0.5 %)	1646 (0.3 %)	3999 (0.4 %)
Eating disorders (F51)	459 (0.1 %)	897 (0.2 %)	1356 (0.1 %)
Personality disorders (F60-F69)	653 (0.1 %)	752 (0.1 %)	1405 (0.1 %)

International Classification of Diseases, tenth revision code range of each diagnostic category is shown in parenthesis.

**Table 3:** Excess life years lost associated with mental health diagnoses by sex and cause of death.

Disorder type	Sex	Cause of death			
		
		All	Natural	Unnatural	Unknown

Any mental health diagnosis	Men	3.83 [3.58; 4.10]	3.42 [3.17; 3.70]	0.45 [0.32; 0.59]	−0.04 [−0.11; 0.03]
	Women	2.19 [1.97; 2.41]	1.94 [1.73; 2.15]	0.22 [0.15; 0.28]	0.03 [−0.02; 0.09]
Organic disorders	Men	11.49 [10.91; 12.01]	10.94 [10.26; 11.60]	0.62 [0.23; 1.11]	−0.07 [−0.21; 0.08]
	Women	9.98 [9.45; 10.61]	9.32 [8.72; 9.97]	0.47 [0.19; 0.75]	0.19 [0.01; 0.40]
Substance use disorders	Men	11.74 [10.35; 13.12]	8.17 [6.67; 9.68]	2.45 [1.48; 3.49]	1.13 [0.45; 1.88]
	Women	9.05 [6.88; 11.19]	7.13 [4.96; 9.26]	1.26 [0.29; 2.35]	0.65 [−0.07; 1.40]
Psychotic disorders	Men	11.10 [9.52; 12.45]	9.69 [7.90; 11.25]	1.38 [0.38; 2.55]	0.03 [−0.36; 0.59]
	Women	9.01 [7.63; 10.41]	8.00 [6.58; 9.50]	0.79 [0.25; 1.37]	0.23 [−0.04; 0.56]
Mood disorders	Men	4.31 [3.98; 4.66]	3.64 [3.28; 3.98]	0.69 [0.51; 0.86]	−0.02 [−0.11; 0.08]
	Women	2.49 [2.19; 2.78]	2.11 [1.83; 2.35]	0.30 [0.21; 0.38]	0.08 [0.02; 0.15]
Anxiety disorders	Men	2.78 [2.43; 3.17]	2.45 [2.10; 2.82]	0.41 [0.25; 0.58]	−0.08 [−0.17; 0.01]
	Women	1.39 [1.12; 1.68]	1.17 [0.90; 1.42]	0.24 [0.16; 0.33]	−0.01 [−0.07; 0.05]
Developmental disorders	Men	7.15 [2.59; 11.46]	5.75 [1.53; 9.84]	1.56 [−1.47; 5.73]	−0.16 [−0.66; 0.85]
	Women	8.51 [4.84; 11.61]	8.83 [5.04; 12.07]	−0.49 [−0.54; −0.45]	0.17 [−0.36; 1.33]
Eating disorders	Men	9.31 [5.38; 12.36]	10.91 [6.99; 13.96]	−1.11 [−1.21; −1.03]	−0.48 [−0.53; −0.44]
	Women	7.84 [4.44; 11.62]	6.21 [3.09; 9.64]	1.76 [−0.26; 3.98]	−0.14 [−0.30; 0.19]
Personality disorders	Men	5.13 [1.16; 8.73]	4.59 [0.70; 8.32]	1.03 [−0.69; 3.07]	−0.49 [−0.53; −0.45]
	Women	2.73 [−0.32; 5.86]	1.83 [−1.12; 4.92]	1.17 [−0.32; 2.92]	−0.27 [−0.30; −0.24]
Alcohol use disorders	Men	11.50 [9.79; 13.07]	8.16 [6.33; 9.66]	2.49 [1.41; 3.60]	0.84 [0.13; 1.70]
	Women	10.24 [7.30; 13.09]	9.02 [6.40; 11.90]	1.25 [0.05; 2.76]	−0.03 [−0.29; 0.59]
Drug use disorders	Men	12.32 [9.69; 15.13]	8.76 [6.13; 11.70]	2.05 [0.58; 3.77]	1.52 [0.28; 2.84]
	Women	7.89 [5.05; 11.06]	4.94 [2.08; 8.17]	1.85 [0.34; 3.69]	1.10 [−0.03; 2.40]
Bipolar disorders	Men	5.21 [4.12; 6.16]	3.61 [2.56; 4.64]	1.52 [0.90; 2.17]	0.08 [−0.23; 0.41]
	Women	4.64 [3.82; 5.44]	3.32 [2.51; 4.08]	0.82 [0.48; 1.14]	0.50 [0.24; 0.77]
Any mental health diagnosis	Men	3.83 [3.58; 4.10]	3.42 [3.17; 3.70]	0.45 [0.32; 0.59]	−0.04 [−0.11; 0.03]
	Women	2.19 [1.97; 2.41]	1.94 [1.73; 2.15]	0.22 [0.15; 0.28]	0.03 [−0.02; 0.09]
Organic disorders	Men	11.49 [10.91; 12.01]	10.94 [10.26; 11.60]	0.62 [0.23; 1.11]	−0.07 [−0.21; 0.08]
	Women	9.98 [9.45; 10.61]	9.32 [8.72; 9.97]	0.47 [0.19; 0.75]	0.19 [0.01; 0.40]
Substance use disorders	Men	11.74 [10.35; 13.12]	8.17 [6.67; 9.68]	2.45 [1.48; 3.49]	1.13 [0.45; 1.88]
	Women	9.05 [6.88; 11.19]	7.13 [4.96; 9.26]	1.26 [0.29; 2.35]	0.65 [−0.07; 1.40]
Psychotic disorders	Men	11.10 [9.52; 12.45]	9.69 [7.90; 11.25]	1.38 [0.38; 2.55]	0.03 [−0.36; 0.59]
	Women	9.01 [7.63; 10.41]	8.00 [6.58; 9.50]	0.79 [0.25; 1.37]	0.23 [−0.04; 0.56]
Mood disorders	Men	4.31 [3.98; 4.66]	3.64 [3.28; 3.98]	0.69 [0.51; 0.86]	−0.02 [−0.11; 0.08]
	Women	2.49 [2.19; 2.78]	2.11 [1.83; 2.35]	0.30 [0.21; 0.38]	0.08 [0.02; 0.15]
Anxiety disorders	Men	2.78 [2.43; 3.17]	2.45 [2.10; 2.82]	0.41 [0.25; 0.58]	−0.08 [−0.17; 0.01]
	Women	1.39 [1.12; 1.68]	1.17 [0.90; 1.42]	0.24 [0.16; 0.33]	−0.01 [−0.07; 0.05]
Developmental disorders	Men	7.15 [2.59; 11.46]	5.75 [1.53; 9.84]	1.56 [−1.47; 5.73]	−0.16 [−0.66; 0.85]
	Women	8.51 [4.84; 11.61]	8.83 [5.04; 12.07]	−0.49 [−0.54; −0.45]	0.17 [−0.36; 1.33]
Eating disorders	Men	9.31 [5.38; 12.36]	10.91 [6.99; 13.96]	−1.11 [−1.21; −1.03]	−0.48 [−0.53; −0.44]
	Women	7.84 [4.44; 11.62]	6.21 [3.09; 9.64]	1.76 [−0.26; 3.98]	−0.14 [−0.30; 0.19]
Personality disorders	Men	5.13 [1.16; 8.73]	4.59 [0.70; 8.32]	1.03 [−0.69; 3.07]	−0.49 [−0.53; −0.45]
	Women	2.73 [−0.32; 5.86]	1.83 [−1.12; 4.92]	1.17 [−0.32; 2.92]	−0.27 [−0.30; −0.24]
Alcohol use disorders	Men	11.50 [9.79; 13.07]	8.16 [6.33; 9.66]	2.49 [1.41; 3.60]	0.84 [0.13; 1.70]
	Women	10.24 [7.30; 13.09]	9.02 [6.40; 11.90]	1.25 [0.05; 2.76]	−0.03 [−0.29; 0.59]
Drug use disorders	Men	12.32 [9.69; 15.13]	8.76 [6.13; 11.70]	2.05 [0.58; 3.77]	1.52 [0.28; 2.84]
	Women	7.89 [5.05; 11.06]	4.94 [2.08; 8.17]	1.85 [0.34; 3.69]	1.10 [−0.03; 2.40]
Bipolar disorders	Men	5.21 [4.12; 6.16]	3.61 [2.56; 4.64]	1.52 [0.90; 2.17]	0.08 [−0.23; 0.41]
	Women	4.64 [3.82; 5.44]	3.32 [2.51; 4.08]	0.82 [0.48; 1.14]	0.50 [0.24; 0.77]
Depressive disorders	Men	4.34 [3.98; 4.68]	3.67 [3.30; 4.02]	0.69 [0.48; 0.87]	−0.02 [−0.11; 0.08]
	Women	2.47 [2.17; 2.74]	2.10 [1.83; 2.34]	0.30 [0.22; 0.38]	0.07 [0.00; 0.13]
Generalised anxiety disorders	Men	2.11 [1.39; 2.85]	1.77 [1.08; 2.43]	0.35 [0.03; 0.79]	−0.02 [−0.22; 0.20]
	Women	0.87 [0.40; 1.43]	0.61 [0.14; 1.17]	0.21 [0.03; 0.38]	0.05 [−0.07; 0.17]
Post-traumatic stress disorders	Men	3.61 [2.11; 5.14]	3.07 [1.59; 4.56]	0.54 [−0.01; 1.14]	−0.01 [−0.34; 0.53]
	Women	1.83 [0.64; 3.02]	1.54 [0.51; 2.69]	0.35 [0.05; 0.68]	−0.07 [−0.28; 0.25]

Data are excess life years lost and 95 % confidence intervals.

## Data Availability

Data were obtained from the International epidemiology Databases to Evaluate AIDS–Southern Africa (IeDEA-SA). Data cannot be made available online because of legal and ethical restrictions. To request data, readers may contact IeDEA-SA for consideration by filling out the online form available at https://www.iedea-sa.org/contact-us/. Statistical code is available under https://github.com/IeDEA-SA/LYL.
